# Consideration of Antifungal Coverage in Treating Infections Related to Delayed Esophageal Perforation from Anterior Cervical Spine Hardware

**DOI:** 10.3390/idr16060082

**Published:** 2024-10-23

**Authors:** Kavita Prasad, John Ceremsak, Jean-Nicolas Gallant, Hannah G. Kay, Erin B. Gettler, Benjamin R. Campbell, Catherine R. Carlile, Byron F. Stephens, Sarah L. Rohde, Patty W. Wright, Christina T. Fiske

**Affiliations:** 1 Department of Otolaryngology-Head and Neck Surgery, Vanderbilt University Medical Center, Nashville, TN 37232, USA; john.ceremsak@vumc.org (J.C.);; 2 School of Medicine, Vanderbilt University, Nashville, TN 37235, USA; 3 Division of Infectious Diseases, Department of Medicine, Vanderbilt University Medical Center, Nashville, TN 37232, USA; 4 Department of Orthopedic Surgery, Vanderbilt University Medical Center, Nashville, TN 37232, USA

**Keywords:** esophageal perforation, antifungal, anterior cervical spine, *Candida*

## Abstract

(1) Background/Objectives: Delayed esophageal perforation following anterior cervical (spine) discectomy and fusion (ACDF) is rare but can lead to serious infectious complications. The treatment usually involves hardware explanation and prolonged intravenous antibiotics; however, there are scarce reports about the microbiology of these infections and corresponding targeted therapy. (2) Methods: Patients diagnosed or treated for delayed esophageal perforation after anterior cervical fusion between 2000–2020 at a tertiary medical center were studied. (3) Results: Seven patients with delayed esophageal perforation following ACDF were identified. The most common bacteria isolated included *Streptococcus*, *Haemophilus*, and *Mycobacterium* species. The cultures from five patients grew fungal species, including *Candida albicans* and *C. glabrata*. All the patients received several weeks of broad-spectrum antibiotics, and, notably, 5/7 patients received antifungal therapy targeting *Candida*. (4) Conclusions: Although the incidence of delayed esophageal perforation following ACDF is low, providers should remain aware of this entity due to the serious infectious complications. Most infections are polymicrobial in nature, and providers should consider empiric antifungal coverage specifically targeting *Candida* species when treating patients with this complication.

## 1. Introduction

A common treatment for traumatic and degenerative conditions of the cervical spine is anterior cervical (spine) discectomy and fusion (ACDF), which involves accessing the anterior cervical spine via mobilization of the esophagus, removing damaged tissues, and fusing vertebra with implanted hardware [1,2]. Esophageal perforation is a rare but potentially lethal complication of ACDF, with incidence rates of <0.5% but mortality rates of up to 33% [3]. The perforation can be iatrogenic at the time of surgery or can be delayed from the loosening and migration of the hardware over time [4]. On average, the time from surgery to diagnosis of a delayed esophageal perforation is two years, but perforations due to hardware migration have been diagnosed up to 20 years after surgery [3,4]. The diagnosis of this complication can be particularly challenging due to the nonspecific complaints of dysphagia, odynophagia, and cervicalgia [5]. However, quick identification and treatment are essential as esophageal perforation can cause significant infectious complications ranging from abscess to osteomyelitis, discitis, mediastinitis, and even sepsis [4,6,7,8].

Despite the severity of esophageal perforation after ACDF, there are limited reports of associated infectious complications, including tissue culture results and targeted antimicrobial regimens. The handful of described infections have been polymicrobial and treated with broad-spectrum antibiotics, with few providers including antifungal coverage [9,10,11,12,13,14,15,16]. The objective of this study was to characterize the infectious organisms related to esophageal perforation following ACDF and to describe the different antimicrobial treatment courses used for these patients.

## 2. Cases

The index patient was a 69-year-old female who presented to an outside hospital with complaints of 1 month of increasing dysphagia. Soon after presentation, she became dyspneic, then hypoxic, and required intubation. Computed tomography (CT) of the neck showed malpositioned anteriorly migrated C3–C7 anterior cervical hardware. The hardware had eroded through the retropharynx, and there was nearby soft tissue stranding, edema, and a gas pattern suggestive of infection. She underwent tracheostomy placement followed by hardware removal and closure of the pharyngo-esophageal defect. Intraoperative cultures were obtained and grew *Candida albicans*, *C. glabrata*, and *Lactobacillus rhamnosus*. *C. glabrata* was susceptible to high-dose fluconazole. The infectious diseases service was consulted and recommended intravenous ampicillin, fluconazole, and metronidazole for six weeks. The patient was recovering well at her 2-month follow-up visit.

To expand our understanding of this infectious process, we systematically identified patients with delayed esophageal perforation following ACDF treated at our institution over the past 20 years using International Classification of Diseases (ICD) codes (Supplement S1). Of the 24 patients identified using that methodology, we found 7 patients who had delayed esophageal perforation from anterior cervical fusion and had intraoperative cultures obtained at the time of surgical debridement (Table 1). Abscess specimens underwent gram staining and traditional aerobic and anaerobic culture, with significant isolates undergoing further identification using standard morphologic traits, biochemical testing, and susceptibility testing. Blood cultures underwent further testing with the ePlex^®^ molecular identification system. The majority of patients were male (*n* = 4, 57%), with a median age at esophageal repair of 49 years (range 37–69 years) and a median time from initial ACDF to esophageal perforation of 38 months (range 5–93 months). None of the patients had a history of diabetes. One patient had a history of immunosuppression with systemic glucocorticoids and methotrexate. The median Charlson Comorbidity Index (CCI), which predicts 10-year survival in patients with multiple comorbidities, at presentation was 2 points (range 1–5 points), corresponding to a 10-year survival of 90% [16]. On computed tomography (CT) imaging, all 7 patients had at least one radiographic finding consistent with infection—usually a rim-enhancing abscess and/or vertebral osteomyelitis.

Intraoperative cultures from all 7 patients grew pathogenic organisms. Three patients had preoperative blood cultures drawn, none of which grew any organisms. Thirteen different microorganisms were isolated from the seven cases. Bacteria isolated included *L. rhamnosus*, *Serratia marcescens*, *Pseudomonas aeruginosa*, and various *Streptococcus*, *Haemophilus*, and *Mycobacterium* (sub)species (Table 2). Cultures from 5 patients isolated fungi, including *C. albicans* and *C. glabrata*. Antimicrobial therapy varied among patients, but most patients received broad-spectrum antibiotics, and five patients also received antifungal therapy targeting *Candida* species. Per institutional protocols, patients were treated with empiric antibiotics in cases of exposed implants or hardware even if no bacteria were isolated.

Of those who underwent surgical repair, median post-operative treatment duration was 6.5 weeks (4.5–9). Of note, one patient received an extended 18-month course of therapy for recurrent neck abscesses secondary to an esophageal fistula, and another patient remains on therapy for mycobacterial osteomyelitis with retained hardware. In terms of clinical outcomes, one patient died from complications of his esophageal perforation; the other six patients eventually recovered or remain on treatment.

## 3. Discussion

Due to the low incidence and heterogenous presentation of esophageal perforations after delayed ACDF hardware extrusion, there is a lack of a consensus on the optimal antimicrobial treatment. While the most commonly isolated bacterial species include coagulase-positive Staphylococcus, Pseudomonas, and Streptococcus species [4], broad-spectrum antibiotics are often used due to presumed polymicrobial infections with esophageal flora, including Gram-positive, Gram-negative, and anaerobic bacteria [17,18,19]. Compared to the prior reports, our case review identified similar bacterial organisms, but a high proportion of the cases also included fungal species (*C. albicans* or *glabrata*).

Slightly over half of the published studies on this complication report specific antimicrobial therapy, of which antifungals are inconsistently included. When antifungal therapy was present, the treatment duration was highly variable, ranging from 1 to 12 weeks [9]. In some cases, the treatment duration was determined based on evidence of clinical improvement, including body temperature, white blood cell count, and wound healing [19].

Our study demonstrates that a significant proportion of patients with delayed esophageal perforations from anterior spinal hardware may have Candida species either as the primary cause of infection or as a part of a polymicrobial process. Thus, while broad-spectrum antibiotics are usually included in the management of these patients, providers should also consider empiric treatment with an antifungal agent that targets Candida species. Since diagnoses of esophageal perforations are often delayed due to variable presentations, early and effective antimicrobial treatment is important, and cultures should be obtained at the time of surgical repair to enable targeted antimicrobial selection.

This study has potential limitations. This was a single-center case series with a small sample size. The identification of cases by ICD codes may have led to the inadvertent omission of cases due to coding errors. Additionally, the patients included in this series had a relatively low 10-year mortality prediction based on the median CCI and may not be reflective of all patients presenting with esophageal perforation. Large multi-institutional database studies are warranted. Given that in two of the seven cases only a single organism was isolated, it is essential to determine the true incidence of single-organism infections to narrow the scope of antibiotic treatment and decrease the risk of antibiotic resistance. Additionally, studies examining the long-term impact of prophylactic antifungal administration, particularly in cases where no fungus is identified, must be conducted.

In conclusion, most infections resulting from delayed esophageal perforation due to ACDF hardware failure are polymicrobial, and providers should consider empiric antifungal coverage when treating patients with this complication. Future studies expanding the total patient cohort are necessary to guide recommendations and management.

## Figures and Tables

**Table 1:** Patient characteristics.

Age at perforation, years, median, (IQR)	49 (37–69)
Male, n (%)	4 (57%)
Caucasian, n (%)	7 (100%)
Charlson Comorbidity Index, median (IQR)	2 (1–5)
Diabetes, n (%)	0 (0%)
Immunosuppression use, n (%)	1 (14%)
Cigarette use, n (%)	5 (71%)
Pack years, median (IQR)	23 (10–90)
Indication for initial ACDF, n (%)	
Vertebral fracture/dislocation	3 (43%)
Ankylosing spondylitis	2 (29%)
Cervical spondylomyelopathy	1 (14%)
Degenerative disc disease	1 (14%)
Unknown	1 (14%)
Presenting symptoms at time of ACDF failure and esophageal perforation, n (%)	
Dysphagia	4 (57%)
Dyspnea and/or stridor	4 (57%)
Incisional leakage	4 (57%)
Neck swelling	3 (43%)
Dysphonia	1 (14%)
Fever	1 (14%)
Cervicalgia	1 (14%)
Vomiting	1 (14%)
Anorexia	1 (14%)

IQR = interquartile range; ACDF = anterior cervical (spine) discectomy and fusion.

**Table 2:** Case characteristics, isolated organisms, imaging findings, and antibiotic courses.

Case	Sex	Age at Perforation, y	Time Since Initial Spinal Surgery (Days)	OR Notes	Spinal Hardware Removed?	Isolated Organism(s)	Imaging Findings	Predominant Antibiotic Course	Length of Antibiotic Course	Clinical Outcome
1	F	69	*	Extrusion of entire plate and screws into pharynx, no evidence of perforation	Yes	*C. albicans* *C. glabrata* *L. rhamnosus*	Retropharyngeal soft tissue stranding and edema with scattered gas	Ampicillin Fluconazole Metronidazole	6 weeks	Recovered
2	M	57	1430	Multiple loose screws, perforation present	Yes	*S. constellatus*	Ill-defined retropharyngeal edema	Amoxicillin-clavulanate	3 weeks	Recovered
3	F	41	856	Single loose, extruded screw with perforation present	Yes	Group F *Streptococci* *S. constellatus* *C. albicans* *H. influenzae*	Abscess posterior to esophagus	Vancomycin Ertapenem Fluconazole	6 weeks	Recovered
4	M	49	382	No loose scres	Yes	*H. parainfluenzae* *P. aeruginosa* *L. rhamnosus* *C. glabrata* *S. viridans*	Extensive paravertebral edema; suspected osteomyelitis	Piperacillin-tazobactam Fluconazole	7 weeks	Recovered
5	M	37	2441	No loose screws	Yes	*C. albicans*	Discitis/osteomyelitis; supraclavicular and lung apex abscesses	Vancomycin Ertapenem Fluconazole	11 weeks	Died
6	M	46	2784	No loose screws	Yes	*C. glabrata* *M. mucogenicum* *S. marcescens* alpha *Streptococci*	Posterior paraspinous abscess	Levofloxacin Fluconazole	1.5 years	Recovered
7	F	66	150	*	No	*M. abscessus* subsp. *massiliense*	Osteomyelitis; retropharyngeal abscess	Clofazimine Azithromycin Amikacin Imipenem	>32 weeks	Ongoing treatment

* Data unavailable.

## Data Availability

Data can be provided upon request to the authors.

## Supplementary Materials

The following supporting information can be downloaded at https://www.mdpi.com/article/10.3390/idr16060082/s1, Supplement S1. Methods.
