# Supplementary material for: Consideration of Antifungal Coverage in Treating Infections Related to Delayed Esophageal Perforation from Anterior Cervical Spine Hardware
Source: Infect Dis Rep. 2024 Oct 23;16(6):1030–5. doi: 10.3390/idr16060082 (PMC11503398; doi:10.3390/idr16060082)
Supplement: Supplementary file 1 [file idr-16-00082-s001.zip › idr-3048987-supplementary.pdf]

Supplement S1. Methods

Patients diagnosed or treated for delayed esophageal perforation due to anterior cervical fusion between January 1, 2000 and July 1, 2020 were identified by querying the Vanderbilt University Medical Center (VUMC) Research Derivative (RD), an IRB-approved, searchable database of electronic health records, with the following *International Classification of Diseases* (ICD) codes: ICD-9 codes 530.4, 478.2, and 996 and ICD-10 codes K22.3, S19.85, and T84. A manual review of each patient's medical records was conducted to determine (a) if the patient had ACDF hardware in place, (b) if the patient had a delayed esophageal perforation, and (c) if the patient had intraoperative cultures from their esophageal repair. In accordance with findings from previous studies that the majority of esophageal perforations following anterior cervical fusion are due to a chronic erosive process as compared to iatrogenic intraoperative injury during surgery [1]; a delayed perforation was defined as that occurring more than 30 days from initial spine surgery.

Only confirmed cases of delayed esophageal perforation from anterior cervical fusion with intraoperative cultures from repair surgery were included (n = 7). Of the 24 patients identified with ICD codes, 6 were excluded due to immediate, rather than delayed, perforation (<30 days from initial spine surgery); 1 was excluded due to perforation caused by direct neck trauma; 5 were excluded who did not have clear evidence of esophageal mucosal perforation on preoperative imaging or endoscopy or during the time of surgery; and 5 were excluded who did not have intraoperative cultures from their repair surgery.

References

1. Halani SH, Baum GR, Riley JP, et al. Esophageal perforation after anterior cervical spine surgery: a systematic review of the literature. *J Neurosurg Spine* 2016; **25**(3): 285-91.
